# Methylation Assessment for the Prediction of Malignancy in Mediastinal Adenopathies Obtained by Endobronchial Ultrasound-Guided Transbronchial Needle Aspiration in Patients with Lung Cancer

**DOI:** 10.3390/cancers11101408

**Published:** 2019-09-20

**Authors:** Virginia Leiro-Fernandez, Loretta De Chiara, Mar Rodríguez-Girondo, Maribel Botana-Rial, Diana Valverde, Manuel Núñez-Delgado, Alberto Fernández-Villar

**Affiliations:** 1 Pulmonary Department, Hospital Álvaro Cunqueiro, Vigo Health Area, 36312 Vigo, Spain; maria.isabel.botana.rial@sergas.es (M.B.-R.); Manuel.nunez.delgado@sergas.es (M.N.-D.); alberto.fernandez.villar@sergas.es (A.F.-V.); 2 NeumoVigoI+i Research Group, Vigo Biomedical Research Institute (IBIV), 36312 Vigo, Spain; 3 Department of Biochemistry, Genetics and Immunology, Biomedical Research Center (CINBIO), University of Vigo, 36310 Vigo, Spain; dianaval@uvigo.es; 4 Department of Medical Statistics and Bioinformatics, Leiden University Medical Center, 2300 RC Leiden, The Netherlands; M.Rodriguez_Girondo@lumc.nl; 5 SiDOR Research Group, Biomedical Research Center (CINBIO), University of Vigo, 36310 Vigo, Spain

**Keywords:** DNA methylation, mediastinal and hilar lymph node, staging, diagnosis, lung cancer, biomarker, endobronchial ultrasound, bronchoscopy

## Abstract

The evaluation of mediastinal lymph nodes is critical for the correct staging of patients with lung cancer (LC). Endobronchial ultrasound-guided transbronchial needle aspiration (EBUS-TBNA) is a minimally invasive technique for mediastinal staging, though unfortunately lymph node micrometastasis is often missed by cytological analysis. The aim of this study was to evaluate the predictive capacity of methylation biomarkers and provide a classification rule for predicting malignancy in false negative EBUS-TBNA samples. The study included 112 patients with a new or suspected diagnosis of LC that were referred to EBUS-TBNA. Methylation of *p16/INK4a*, *MGMT*, *SHOX2*, *E-cadherin*, *DLEC1*, and *RASSF1A* was quantified by nested methylation-specific qPCR in 218 EBUS-TBNA lymph node samples. Cross-validated linear regression models were evaluated to predict malignancy. According to EBUS-TBNA and final diagnosis, 90 samples were true positives for malignancy, 110 were true negatives, and 18 were false negatives. *MGMT*, *SHOX2*, and *E-cadherin* were the methylation markers that better predicted malignancy. The model including sex, age, short axis diameter and standard uptake value of adenopathy, and *SHOX2* showed 82.7% cross-validated sensitivity and 82.4% specificity for the detection of malignant lymphadenopathies among negative cytology samples. Our results suggest that the predictive model approach proposed can complement EBUS-TBNA for mediastinal staging.

## 1. Introduction

Lung cancer (LC) is the leading cause of cancer death worldwide [1]. Approximately 80% of the cases are non-small cell lung cancer (NSCLC), with lung adenocarcinoma being the most prevalent type. Correct mediastinal and hilar staging is critical for choosing the best option for management and treatment of patients with NSCLC who are potential candidates of curative therapeutic strategies that include surgery, radiotherapy, chemotherapy and multimodal treatments [2,3]. Endobronchial ultrasound-guided transbronchial needle aspiration (EBUS-TBNA) and endoscopic ultrasound-guided fine needle aspiration (EUS-FNA) are minimally invasive techniques that have shown high diagnostic value for mediastinal staging in patients with LC [4,5]. Sampling of mediastinal lymph nodes with these procedures associates with reduced morbidity compared to surgical biopsy (usually mediastinoscopy or video-assisted thoracoscopy), which is the current gold-standard for mediastinal lymph node staging [6]. It has been demonstrated that samples obtained with EBUS-TBNA are adequate for accurate characterization of cytopathology and molecular testing, including methylation biomarkers, and can have a role in diagnosis, prognosis, and response to chemotherapy [7,8,9,10,11,12].

Epigenetic alterations are known to contribute to tumor development, progression, and metastasis in NSCLC [13]. DNA methylation is one of the most common epigenetic mechanisms studied. Aberrantly methylated genes are attractive candidate markers, as cancer-specific methylation occurs at all stages of tumorigenesis, appears to be stable, yields an amplifiable signal, and can be assayed with high accuracy [14,15]. Promoter hypermethylation plays an important role in the inactivation of tumor suppressor genes, and methylation profiles have been considered promising biomarkers in LC [14,16].

Cytological analysis of lymph node samples obtained with EBUS-TBNA shows sensitivity between 85% and 100% for the detection of lymph node malignancy [17,18,19]. However, metastatic involvement cannot be ruled out in all negative cytological adenopathies, reporting negative predictive values (NPV) ranging from 11–97%. The proportion of negative cytological samples that are actually false negatives result in incorrect staging, and in cases with high suspicion of metastasis, surgical staging is required. Methylation analysis has the potential to increase the diagnostic performance of cytology in EBUS-TBNA samples.

In this study, we selected six tumor suppressor genes that have been reported to be inactivated by promoter hypermethylation in NSCLC. The genes analyzed are involved in important cellular functions: *p16/INK4a* is a key regulator of the cell cycle and has been associated with poor prognosis in NSCLC patients [20]; *MGMT* codifies a critical enzyme that repairs DNA alkylation damages and its hypermethylation is associated with an increased risk of NSCLC and is more prevalent in advanced stages [21,22]; *SHOX2* has DNA-binding transcription factor activity and has been described as a valuable biomarker for LC diagnosis and staging [23,24]; *E-cadherin* has a key role in cell-cell adhesion and tissue differentiation, and its hypermethylation contributes to cancer progression [25]; *DLEC1* is implicated in cell proliferation and differentiation, and is frequently methylated in LC lymphatic metastasis [26,27]; and *RASSF1A* is involved in the regulation of apoptosis and its methylation is associated with poor survival [28,29].

The aim of our study was to analyze methylation in *p16/INK4a*, *MGMT*, *SHOX2*, *E-cadherin*, *DLEC1*, and *RASSF1A* in EBUS-TBNA samples, and determine their ability to detect metastatic infiltration (micrometastasis). We also evaluated the predictive capacity of clinical and epidemiological variables, and together with methylation biomarkers, provide a classification rule for predicting malignancy in negative cytological samples (false negatives).

## 2. Results

### 2.1. Clinical Characteristics of Patients and Lymph Nodes

We included in our study 218 lymph node samples obtained from 112 patients. Epidemiological and clinical data from patients and adenopathies are summarized in Table S1 and Table 1, respectively. Men comprised 83.9% of the study cohort, and the mean age was 64.92 ± 9.89 years. The most frequent location of the primary tumor was the upper right lobe, and adenocarcinoma was the most prevalent histology. The most frequent lymph node station aspiration sites were 4R (36.2%) and subcarinal (33.9%). According to cytology, 90 samples (41.3%) were positive for malignancy (true positives, TP). Among the 128 negative for malignancy lymph node samples, 18 (8.2%) were found to be malignant (false negative results, FN). Among the 18 FN, malignancy was surgically confirmed in 9 lymph nodes, while the other 9 cases experienced adenopathy growth during follow-up. Out of these, malignancy was confirmed by a new EBUS-TBNA in three cases, and in the other six cases the diagnosis of malignancy was assumed by the LC Multidisciplinary Committee. Therefore, the prevalence of malignant lymph nodes in this cohort was 49.5% (108 samples).

Patients with lymph node metastasis (men vs. women: 77.3% vs. 22.7%) and non-metastatic lymph nodes (men vs. women: 93.5% vs. 6.5%) were more frequently men (*p* = 0.034) (Table 2). Metastatic lymph nodes had a greater short axis diameter (*p* = 0.065) and a greater SUV in PET scanning (*p* = 0.046) compared to non-metastatic lymph nodes. We also found that the difference between SUVs from the tumor and adenopathy was increased in non-metastatic lymph nodes (*p* = 0.172).

### 2.2. Methylation of Candidate Genes and Evaluation of the Diagnostic Performance

**Figure 1:**
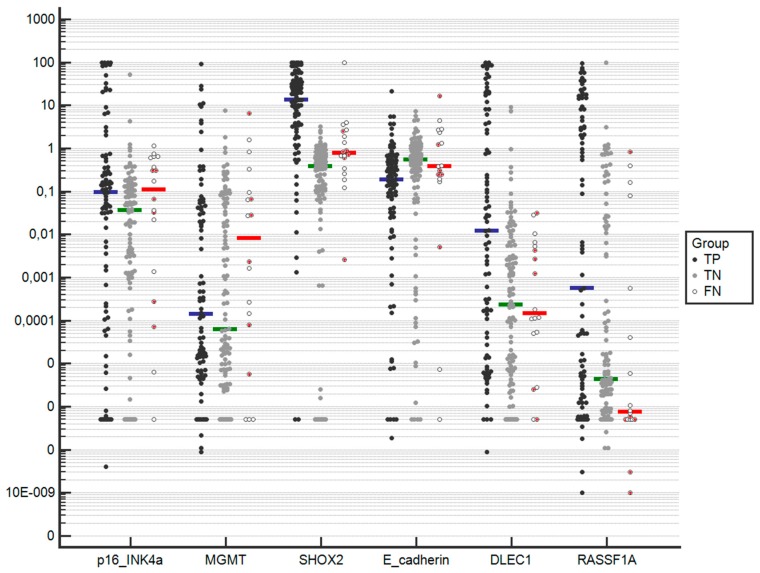
Representation of the methylation percentage of the six candidate genes analyzed, grouped according to EBUS-TBNA and final diagnosis. TP: true positive; FN: false negative; TN: true negative. Median methylation is represented with a horizontal blue line for TP, a green line for TN, and a red line for FN. The six FN patients in which malignancy was not histologically confirmed are represented as filled red circles. In the y-axis, Please change 10E-009 into scientific notion 1.0×10^−9^.

The six methylation candidate genes *p16/INK4a*, *MGMT*, *SHOX2*, *E-cadherin*, *DLEC1*, and *RASSF1A* were analyzed in all the lymph node samples. Mean, median, and IQ range is shown in Table 3 according to the cytology result and the final diagnosis. The dot plot in Figure 1 represents the methylation values for each gene and group. In general, TP showed a larger mean/median for all genes in relation to the other groups. Comparison of methylation values between TP, FN, and TN resulted in statistically significant differences for all candidates, except *MGMT*.

When we compared samples with negative cytology (TN vs. FN), differences in methylation were only found for *SHOX2*, observing increased values in FN, and suggesting its utility for the detection of malignancy. The area under the curve (AUC) for all methylated genes is provided in Table 3 for the diagnosis of malignancy based on all samples, and for negative cytology cases (FN vs. TN). Overall, the marker that better performed in both contexts was *SHOX2*, with an AUC = 0.862 (95% CI 0.809–0.905) for detecting malignancy in all lymph node samples, and an AUC = 0.732 (95% CI 0.646–0.806) in the case of negative cytology samples. Methylated *DLEC1* showed the second best AUC when all samples were included, though its performance was the worst for negative lymph nodes. The individual performance of the six methylated genes (sensitivity and specificity based on the Youden index) is provided in Table S2, indicating that the diagnostic yield of the individual methylation markers is not optimal for the detection of malignancy.

### 2.3. Performance of A Multivariate Model to Predict Malignancy in Negative Lymph Node Samples

Epidemiological, clinical, and methylation variables available for lymph node samples were first evaluated in an univariate analysis limited to negative cytology samples, with the aim of determining the importance of each variable for predicting malignancy. Table 4 summarizes these analyses, where we only included variables that are possible determinants for malignancy in cytology negative lymph node samples.

**Figure 2:**
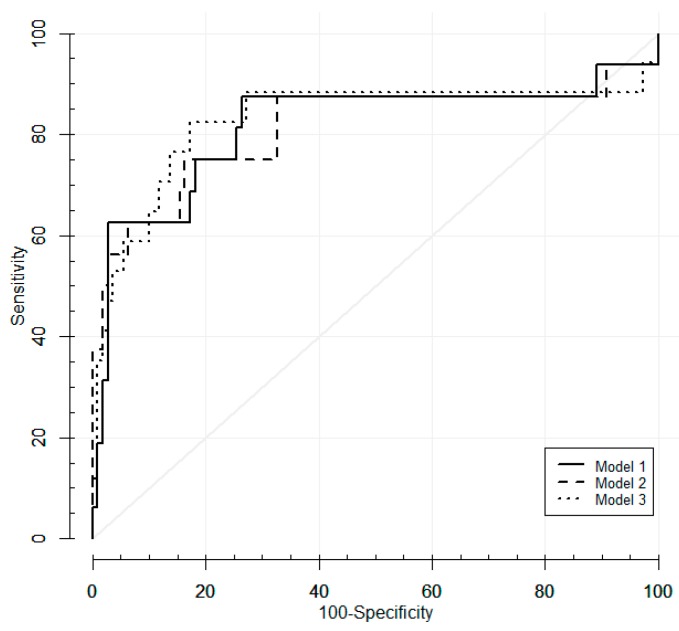
ROC curves of the cross-validated predicted probabilities of the three models for the prediction of malignancy in cytological negative lymph nodes.

According to the criteria for variable selection (*p*-value < 0.15), the epidemiological variables with the largest associations with diagnosis of malignancy were sex and age; the clinical variables most associated with diagnosis were short axis diameter of adenopathy and SUV of adenopathy, while the strongest associations among the methylation variables were found for *MGMT*, *SHOX2*, and *E-cadherin*. Based on these results, we built multivariate predictive models and evaluated its performance in terms of AUCs using 10-fold cross-validation (Table 5 and Figure 2).

The three models included the two epidemiological and clinical variables; additionally, Model 1 included the three methylation genes (*SHOX2*, *E-cadherin*, and *MGMT*), Model 2 included the two genes that were the most associated with diagnosis (*SHOX2* and *E-cadherin*), while *SHOX2* was the only methylation marker included in Model 3. After 10-fold cross-validation, these three prediction models showed similar AUCs, over 0.80. Based on the Youden index, the model that best performed was Model 3, which included the following variables: sex, age, short axis diameter of adenopathy, SUV of adenopathy, and methylated *SHOX2*. This model showed a cross-validated specificity of 82.7% and 82.4% sensitivity for the detection of malignant lymphadenopathies among negative cytology samples, with a 96.8% negative predictive value and a 42.4% positive predictive value.

## 3. Discussion

Mediastinal lymph nodes are the most common sites of metastasis, so an accurate evaluation is a critical component for correct staging of NSCLC patients. The EBUS-TBNA examination is considered a key tool in clinical practice guidelines on mediastinal diagnosis and staging, with sensitivity ranging from 88–91% [17,18,30,31]. Various studies have shown that, even when changes are observed on CT or PET, the reliability of negative EBUS-TBNA results varies widely, depending on a wide range of variables related to the tumor (type, site, stage, and size), the lymphadenopathies (site, echographic features, size, and PET avidity), the procedure (number of passes, number and location of the stations sampled, and type of sedation), the experience of the endoscopist and pathologist, and the quality of the sample obtained [32,33]. Thus, the main problem is making the convenient decision of confirming negative results by surgery [5]. Consequently, micrometastasis may not be diagnosed because a complete analysis of the adenopathy is not performed.

In this study, the prevalence of malignant lymph nodes was 49.5% and the sensitivity of EBUS-TBNA was 83.3%, with a negative predictive value of 86%, which is similar to the ASTER randomized controlled trial [5]. Since molecular testing has the potential of detecting a very small number of cancer cells (micrometastasis), we hypothesized that the use of methylation biomarkers combined with other factors could improve the sensitivity of EBUS-TBNA, which supports the likelihood of benign results. The six genes analyzed in our study are involved in important cellular functions, and aberrant methylation has been reported in LC patients [20,21,22,23,24,25,26,27,28,29]. The viability of quantifying methylation in EBUS-TBNA samples was confirmed in previous studies [9,10].

In terms of median methylation, *p16/INK4a*, *MGMT*, *SHOX2*, *DLEC1*, and *RASSF1A* were found hypermethylated in TP malignant lymph nodes compared to TN non-malignant lymph nodes, while *E-cadherin* was the only gene found hypomethylated. However, when the analysis was restricted to the negative samples (TN and FN), the latter group showed hypermethylation in *p16/INK4a*, *MGMT*, and *SHOX2*, whereas *E-cadherin*, *DLEC1* and *RASSF1A* were hypomethylated. Among the six genes analyzed, *SHOX2* exhibited the highest AUC and was the gene that better predicted malignancy among negative EBUS-TBNA samples. Methylated *SHOX2* has been studied previously for detecting LC in lymph node samples obtained by EBUS-TBNA [24], in bronchial aspirates, pleural effusion, plasma, and tumor tissue [34]. A meta-analysis exploring *SHOX2* methylation for LC diagnosis in different samples estimated a pooled sensitivity of 70% with 96% specificity (AUC = 0.96), supporting its value for confirming benignity for negative results [34].

In relation to the other candidate genes, though *E-cadherin* and *MGMT* apparently did not show an optimal performance for the detection of malignancy in terms of AUC, the univariate analysis revealed their capacity for predicting malignancy in negative lymph nodes. E-cadherin protein is an epithelial marker of the epithelial-mesenchymal transition (EMT) process and acts as a tumor suppressor in tumor metastasis, epigenetically regulated [35]. Recently, it was demonstrated that the reduced expression of E-cadherin is related to SIX2 overexpression, which promotes NSCLC cell stemness, resulting in metastasis [36]. On the other hand, *MGMT* hypermethylation has been largely reported in tissue and other samples from NSCLC patients and is found more frequently methylated in Stage III and IV tumors, which suggests an increased ability of proliferation and invasion of tumor cells [22].

As reported in other studies [32,33,37] and confirmed in our work, patient’s age, lymph node size, and SUV uptake are factors associated with increased likelihood of malignancy of lymphadenopathies. The combination of these variables and sex, together with the methylation biomarkers *MGMT*, *E-cadherin*, and *SHOX2*, improved the detection of lymphatic micrometastases in negative conventional evaluation. The decision rule that showed the largest accuracy was Model 3 and included *SHOX2*, patient’s sex and age, lymph node size, and SUV (cross-validated AUC = 0.827). This prediction model seems to outperform the other two models in terms of both sensitivity and specificity. The optimal cut-off point for Model 3 (*p* score > 0.076; based on the Youden index) correctly classified 82.4% of the malignant lymphadenopathies that were missed by EBUS-TBNA (FN). On the other hand, the model correctly ruled out malignancy in 82.7% of the cytological negative samples, misclassifying as malignant 17.3% of the non-metastatic samples.

According to the cross-validated decision rule and based on our subcohort, 15 of the 18 FN have a positive result (TP for the model), while 19 of the 110 TN showed a positive result (FP for the model). A diagnostic confirmation should be completed in all these cases that score above the established cut-off. In terms of the NPV, the probability that a patient with a negative result (*p* score below the cut-off) truly has no metastasis on lymph node corresponds to 96.8%. In our opinion, when wanting to rule out malignancy in the diagnosis and/or non-invasive mediastinal staging of LC, a highly NPV is desirable among EBUS-TBNA negatives. The classification rule proposed in our study seems useful for clinical decision-making in the management of patients with a negative EBUS-TBNA.

With the intention of facilitating the understanding and interpretation of the predictive algorithm proposed, Table S3 shows the values of the five variables that make up the predictive model for six patients. The diagnosis according to Model 3 and the final clinical diagnosis of the six cases are provided in the table. Among these cases, malignancy would not be detected in Patient 3, while Patient 5 would be subjected to unnecessary invasive procedures.

Other molecular biomarkers have been proposed for EBUS-TBNA-based mediastinal staging [38]. Expression of *p53*, *K-ras* [39], and *lunx* [40] has shown to improve the detection of occult lymph node metastasis. Human telomerase catalytic subunit gene (*hTERT*) was also suggested as a biomarker for detecting micrometastasis [38]. More recently, expression of miRNAs was also assessed for molecular staging of nodes using EBUS-TBNA samples [41]. Among the five candidates evaluated in 39 malignant and 11 benign lymph nodes, miR-200c showed the highest diagnostic yield, resulting in a 97.4% sensitivity, an 81.8% specificity, and a 90.0% NPV. Restaging of FFPE EBUS-TBNA samples from 10 patients (4 EBUS-TBNA FN included) rendered a 100% sensitivity, a 60% specificity, and a 100% NPV.

This study has some limitations. First, 9 of the 18 patients in the EBUS-TBNA false negative group showed progression on image techniques, but 6 of them had no histological confirmation of malignancy. Tissue confirmation is the reference standard recommended by the European Society of Thoracic Surgeons [31], though in clinical practice, not all patients can undergo surgery for malignancy confirmation. Second, the small sample size, especially in the false negative group, leads to high uncertainty in the model development, which limits its predictive performance. Third, this study was conducted in a single center with a small sample size, so the results must be confirmed in a multicenter study including a larger number of EBUS-TBNA samples.

## 4. Materials and Methods

### 4.1. Patients and Study Design

The study included 112 patients with a new or suspected diagnosis of LC that met the criteria for mediastinal study for diagnosis or staging proposes and that were referred to EBUS-TBNA. CT scanning and PET-CT scanning were used to reach a presumptive TNM stage. All results were reviewed at multidisciplinary LC team meetings with week periodicity. All patients’ medical records are included in an electronic clinical history that belongs to the National Care Service. Patients without an evaluable sample according to the cytologist, or an insufficient sample for further methylation studies were excluded. All patients were recruited at Pulmonary Department from Complexo Hospitalario Universitario de Vigo. The study was conducted according to the clinical and ethical principles of the Spanish Government and the Declaration of Helsinki and was approved by the Ethics Committee for Clinical Research of Galicia (2009/133). Informed consent was obtained from all patients.

### 4.2. Study Interventions

Most of the procedures were ambulatory and took place in a conventional bronchoscopy room. A bronchoscope model BF-UC180F-OL8 (Olympus, Tokyo, Japan) and ultrasound equipment Aloka ProSound Alpha 5 (Hitachi-Aloka, Tokyo, Japan) were used. Lymph nodes were classified according to the International Association for the Study of Lung Cancer lymph node map [42]. Lymph nodes were considered positive if they were >1 cm in short axis on the CT scan or had an SUV (standard uptake value) >2.5 on PET-TC; lymph nodes measuring >5 mm by EBUS were sampled, even if they were normal on CT and/or PET-CT. After endoscopic examination, each node was measured and sampled using a NA2015X-4022 needle (Olympus, Tokyo, Japan). Depending on the immediate results obtained, more than one pass was made. Rapid on-site evaluation (ROSE) was performed by an expert cytologist during the procedure. The material was recovered, and the sample was fixed in alcohol and immediately examined by a cytologist. Diff–Quick staining of the cytological sample was performed in situ, and Papanicolaou staining was performed later. Cytology obtained during EBUS-TBNA consists mainly of “loose” cells or small groups of cells. While in the majority of cases the final diagnosis of malignancy is based on the Diff–Quick slides, nuclear details are clear in the Papanicolaou smears and allow a better identification of malignant cells when the quality of the Diff–Quick slides is not optimal. These two methods complement each other and were used.

The cytologist confirmed the adequacy of the sample and classified the material as follows: a normal node (predominantly lymphoid cells without atypia and/or anthracotic material—negative cytology); a node with neoplastic infiltration (a presence of neoplastic lymph node cells and cellularity—positive cytology); or a non-evaluable sample (a presence of only blood or bronchial cells). A representative portion of the sample was resuspended in sterile saline solution and immediately frozen at −20 °C for subsequent methylation analyses.

In the case of negative mediastinal staging results following EBUS and high clinical suspicion, all medically acceptable patients were referred to a confirmatory surgical biopsy (cervical mediastinoscopy or video-assisted thoracoscopic surgery). Patients who did not undergo confirmation by surgery were followed up for at least one year. If the lymph node did not grow during surveillance, the results were considered negative. However, if the lymph node grew during surveillance, the patient was evaluated by the LC Multidisciplinary Committee to make a decision about considering re-biopsy by non-surgical pathological staging, or to assume the diagnosis of malignancy when adequate clinical context was present according to the high risk of regional extension. All doubtful cases were excluded.

According to cytological analysis and clinical evaluation, each sample was classified as true-positive (TP, when cytology was positive and metastasis was clinically confirmed), true-negative (TN, when cytology was negative and metastasis was not evidenced after surgery or no modification in lymph node size was observed during 1 year surveillance), and false-negative (FN, when cytology was negative but metastatic lymph node infiltration was evidenced after surgery or significant growth of lymph node was detected during follow-up).

### 4.3. DNA Extraction and Sodium Bisulfite Modification

DNA was extracted from cytological lymph node samples and eluted with 50 µL of warmed-water (QIAamp DNA Blood Mini Kit, Qiagen, Hilden, Germany). DNA was quantified using a NanoDrop 2000 c (Thermo Scientific, Waltham, MA, USA). Mean DNA concentration resulted in 103.1 ng/mL, and 81.5% of the samples showed a 260/280 ratio of ~1.8. DNA was aliquoted and stored at −20 °C until used.

Sodium bisulfite modification was performed using EZ DNA Methylation-Direct kit (Zymo Research, Irvin, CA, USA). Briefly, 20 μL of DNA were bisulfite-modified according to the manufacturer´s instructions, and finally eluted in 20 μL. Modified DNA was stored at −80 °C until used.

A fully methylated control was prepared from DNA extracted from peripheral blood mononuclear cells and treated with CpG methyltransferase (M.SssI; New England Biolabs, Ipswich, MA, USA). This fully methylated control was prepared in large amounts and was used in all the methylation analyses. An unmethylated control, not treated with M.SssI, was included in each bisulfite treatment and was also included in all the analyses. DNA extraction and methylation analyses were performed blinded to the cytology result.

### 4.4. Methylation Analysis of the Candidate Genes

Methylation of *p16/INK4a*, *MGMT*, *SHOX2*, *E-cadherin*, *DLEC1*, and *RASSF1A* was assessed using a nested methylation-specific qPCR approach. In the first-step PCR (pre-amplification), a methylation-independent product was amplified for each gene using outer primers (Table S4). PCR was performed in a 25 µL reaction mix containing 2 µL of bisulfite-modified DNA, 0.72 µM forward and reverse outer primers, a 75 µM dNTPs mixture, a 1× Ex Taq Buffer, and 1 unit of Takara Ex Taq HotStart, with the following cycling conditions: 95 °C for 5 min, 32 cycles of 95 °C for 30 s, 30 s at the appropriate temperature for each amplicon, 72 °C for 30 s, and finally 72 °C for 7 min. A fully methylated control, an unmethylated control, a 1/10 dilution of the fully methylated control, and a no-template control were always included in each PCR.

In the second-step, an MS-qPCR was performed using a 1/300–1/500 dilution of the previous PCR product. Real-time PCR was carried out in triplicate in a 20 µL volume containing 2 µL of the diluted PCR, 600–1.000 nM of each primer, 200 nM of probe, and 1× TaqMan Universal PCR Master Mix No AmpErase UNG (Applied Biosystems, Waltham, MA, USA), with an annealing temperature of 60–62 °C during 40 cycles. Primers, probes, and MS-qPCR amplification efficiencies are shown in Table S3 for each gene. Amplifications were carried out in 48-well plates and run on a StepOne instrument (Applied Biosystems). In each plate, dilutions of the previously amplified fully methylated control (100%, 10% and 1%), unmethylated control, 1/10 diluted fully methylated control, and no-template control were always included, besides samples and qPCR no-template control.

The methylation-independent amplification of the *MYOD1* gene was used to normalize for DNA input, using the same two-step nested PCR approach.

### 4.5. Analysis of the MS-qPCR Data

MS-qPCR data for each of the studied genes was derived from 5 independent assays (standard curve) consisting of 10-steps dilutions (100%, 75%, 50%, 25%, 10%, 5%, 1%, 0.5%, 0.25%, and 0.1% methylation) of the fully methylated control. The non-normalized methylation percentage (NNMP) of each sample and gene was estimated from a linear fit of the mean Cq (quantification cycle) as a function of the log10 methylation percentage. Since DNA concentration varied among samples, MYOD1 was used to normalize. To estimate DNA quantity (DNAQ) of each sample, we applied the same dilution procedure over MYOD1, given that the region analyzed does not contain CpG dinucleotides, and amplification is therefore methylation-independent. Finally, the normalized methylation percentage (NMP) was calculated as follows:

(1) $$NMPsample\;\left(gene\right)=\frac{NNMPsample\;\left(gene\right)}{DNAQsample\;\left(MYOD1\right)}\times 100$$

### 4.6. Statistical Analysis

Categorical variables were expressed as frequencies, while continuous variables were expressed as mean and standard deviation (SD) or median and IQ range. The chi-square test and Fisher’s exact test were used to compare frequencies between metastatic and non-metastatic lymph nodes. Kruskal–Wallis and Mann–Whitney U tests were used to compare methylation according to cytology result and final diagnosis. Univariate analysis was performed to determine if variables were predictive for malignancy. Multivariate logistic regression models were used to find a predictive model for the dependent variable final diagnosis of malignancy, including epidemiological, clinical, and methylation variables with a univariate p-value inferior to 0.15. Multivariate logistic regression models were 10-fold cross-validated to internally validate their performance and help to protect from overfitting.

Diagnostic performance was analyzed using ROC curves and the AUC (area under the curve) was reported. Sensitivity and specificity were calculated based on the Youden index, in addition to the predictive values, which were based on the prevalence of malignancy among negative cytological samples in our cohort. For the multivariate logistic regression models, AUC and the diagnostic parameters were based on the event predicted probabilities of each model obtained with 10-fold cross-validation. The analyses were performed with SPSS 21.0 (IBM Corporation, Armonk, NY, USA), MedCalc statistical software v19.0.6 (Ostend, Belgium) and R program package (R Foundation for Statistical Computing, Wirtschafts Universität, Wien, Austria). Data were analyzed using two-sided tests; *p* values < 0.05 were considered significant.

## 5. Conclusions

The evaluation of *SHOX2* methylation in EBUS-TBNA samples combined with the sex and age from the patient, and the diameter and SUV of the lymphadenopathy, increases the accuracy of EBUS-TBNA for the diagnosis of metastatic node involvement in NSCLC. The use of this predictive model tool can allow a more adequate selection of patients requiring surgical confirmation in the presence of a negative cytological result.

## Figures and Tables

**Table 1:** Characteristics of lymph nodes.

Adenopathy (*n* = 218)	*n* (%)
Mediastinal Hilar	186 (85.3%) 32 (14.7%)
**Adenopathy**	
2R, 2L, 4R, 4L, 7 10, 11, 12 8, 9	182 (83.5%) 32 (14.7%) 4 (1.8%)
**Adenopathy Short Axis (mm), mean ± SD**	12.1 ± 4.9
**Number of Punctures, mean ± SD**	1.9 ± 1.1
**SUV Adenopathy, mean ± SD**	4.2 ± 3.1
**Metastatic Node**	
Adenocarcinoma Squamous cell Large cell	71 (32.6%) 29 (13.3%) 8 (3.6%)
**EBUS-TBNA Results**	
Metastatic nodes (true positives, TP) Negative (true + false negatives, TN + FN) Non metastatic nodes (true negatives, TN) Metastatic nodes (false negatives, FN)	90 (41.3%) 128 (58.7%) 110 (50.5%) 18 (8.2%)

SUV: standard uptake value.

**Table 2:** Comparison of patients and lymph nodes characteristics according to EBUS-TBNA and final diagnosis.

Variable (%), mean ± SD	Metastatic Lymph Node	Non-Metastatic Lymph Node	*p*-Value
Sex, male	51 (77.3%)	43 (93.5%)	0.034
Age, mean ± SD	66.0 ± 10.7	63.1 ± 8.5	0.754
Tobacco habit, actual or former smoker	57 (86.4%)	43 (93.5%)	0.358
Tumor diameter (mm), mean ± SD	34.5 ± 18.2	36.7 ± 22.7	0.681
SUV Tumor, mean ± SD	10.2 ± 5.1	10.69 ± 6.4	0.423
Adenopathy location, ipsilateral	56 (84.8%)	38 (82.6%)	0.254
Adenopathy short axis (mm), mean ± SD	13.3 ± 5.5	11.4 ± 5.0	0.065
SUV Adenopathy, mean ± SD	6.0 ± 3.9	2.7 ± 1.3	0.046
Ratio SUVa/SUVt, mean ± SD	0.7 ± 0.7	0.5 ± 0.6	0.391
Difference SUVt-SUVa, mean ± SD	4.5 ± 5.4	8.1 ± 6.6	0.172

SUV: standard uptake value.

**Table 3:** Normalized methylation percentages of candidate genes according to EBUS-TBNA and final diagnosis.

Gene	TP (*n* = 90)	FN (*n* = 18)	TN (*n* = 110)	*p*-Value^1^	AUC^1^	AUC^2^
	NMP Mean	NMP Mean	NMP Mean	*p*-Value^2^	(95% CI)	(95% CI)
	NMP Median	NMP Median	NMP Median		(108 vs. 110)	(18 vs. 110)
	(IQ range)	(IQ range)	(IQ range)			
*p16/INK4a*	9.69	0.29	0.62		0.603	0.629
	0.09	0.12	0.04	0.030	(0.535–0.668)	(0.539–0.713)
	(2 × 10^−4^–0.60)	(1 × 10^−3^–0.63)	(9 × 10^−4^–0.13)	0.079		
*MGMT*	2.10	0.53	0.14		0.542	0.611
	1 × 10^−4^	0.02	6 × 10^−5^	0.302	(0.474–0.610)	(0.521–0.696)
	(7 × 10^−6^–0.04)	(6 × 10^−5^–0.15)	(4 × 10^−6^–0.05)	0.131		
*SHOX2*	25.26	6.75	0.49		0.862	0.732
	13.52	0.76	0.39	<0.0001	(0.809–0.905)	(0.646–0.806)
	(2.73–34.37)	(0.32–2.58)	(0.10–0.70)	0.002		
*E-cadherin*	0.76	1.97	0.80		0.602 ^3^	0.531
	0.19	0.38	0.54	0.006	(0.533–0.667)	(0.440–0.620)
	(0.03–0.56)	(0.18–2.52)	(0.13–1.01)	0.681		
*DLEC1*	11.07	0.005	0.17		0.655	0.521
	0.01	1.5 × 10^−4^	2×10^−4^	<0.0001	(0.581–0.718)	(0.431–0.610)
	(2 × 10^−5^–4.04)	(0–0.032)	(4 × 10^−6^–6 × 10^−3^)	0.773		
*RASSF1A*	8.28	0.08	1.04		0.575	0.613 ^3^
	6 × 10^−4^	8 × 10^−7^	4 × 10^−6^	0.003	(0.505–0.642)	(0.523–0.697)
	(6 × 10^−7^–5.30)	(0–0.02)	(9 × 10^−7^–0.014)	0.126		

TP: true positives; FN: false negatives; TN: true negatives; NMP: Normalized methylation percentage; *p*-Value^1^: Kruskal–Wallis test for comparing TP, FN, and TN; *p*-Value^2^: Mann–Whitney U test used to compare FN and TN; AUC^1^: area under the curve for the diagnosis of malignancy (TP and FN vs. TN); AUC^2^: under the curve for the diagnosis of malignancy (FN vs. TN). ^3^: a lower result in the test (less methylation) implicates a more positive test.

**Table 4:** Univariate analysis of epidemiological and clinical variables, and methylation candidates in cytological negative samples according to EBUS-TBNA and final diagnosis.

Variable	TN	FN	OR (95% CI)	*p*-Value
Sex, male	103 (93.6%)	11 (61.1%)	9.36 (2.8–31.7)	<0.001
Age, mean ± SD	63.3 ± 8.8	70.3 ± 9.6	1.09 (1.027–1.156)	0.004
Location of primary tumor, UL	78 (70.9%)	14 (77.8%)	0.67 (0.22–2.07)	0.488
Tumor histology, adenocarcinoma	44 (60.3%)	6 (46.2%)	0.55 (0.17–1.85)	0.346
Tumor diameter (mm), mean ± SD	34.9 ± 19.8	40.8 ± 20.2	1.01 (0.99–1.04)	0.241
SUV primary tumor, mean ± SD	11.3 ± 6.3	13.2 ± 3.9	1.05 (0.97–1.14)	0.234
Adenopathy short axis (mm), mean ± SD	11.5 ± 4.7	9.7 ± 2.7	0.9 (0.788–1.028)	0.120
SUV adenopathy, mean ± SD	2.9 ± 1.5	4.0 ± 2.4	1.37 (1.04–1.79)	0.025
*p16/INK4a*, mean ± SD	0.6 ± 5.04	0.3 ± 0.3	0.976 (0.82–1.17)	0.791
*MGMT*, mean ± SD	0.1 ± 0.7	0.5 ± 1.5	1.35 (0.91–2.01)	0.142
*SHOX2*, mean ± SD	0.5 ± 0.5	6.8 ± 23.3	2.90 (1.56–5.39)	0.001
*E-cadherin*, mean ± SD	0.8 ± 1.1	2.0 ± 4.0	1.27 (0.98–1.64)	0.068
*DLEC1*, mean ± SD	0.2 ± 1.1	5 × 10^−3^ ± 1 × 10^−2^	0 (0–0)	0.529
*RASSF1A*, mean ± SD	1.0 ± 9.5	0.1 ± 0.2	0.638 (0.10–3.98)	0.631

TN: true negative; FN: false negative; OR: odds ratio; UL: upper lobe; SUV: standard uptake value.

**Table 5:** Multivariate regression models for the detection of malignancy in cytological negative lymph nodes.

Model	Variables Included	Apparent AUC	AUC	Specificity ^1^	+PV ^1^	Cut-Off ^1^
		(95% CI)	10-fold CV	Sensitivity ^1^	−PV ^1^	
**1**	Sex, age, adenopathy short axis, SUV of	0.958	0.815	73.6%	32.6%	>0.021
	adenopathy, *MGMT*, *SHOX2*, *E-cadherin*	(0.907–0.986)		87.5%	97.6%	
**2**	Sex, age, adenopathy short axis, SUV of	0.953	0.812	83.6%	40.0%	>0.066
	adenopathy, *SHOX2*, *E-cadherin*	(0.900–0.983)		75.0%	95.8%	
**3**	Sex, age, adenopathy short axis, SUV of	0.951	0.827	82.7%	42.4%	>0.076
	adenopathy, *SHOX2*	(0.897–0.981)		82.4%	96.8%	

SUV: standard uptake value; AUC: area under the curve; CV: cross-validated; +PV: positive predictive value; −PV: negative predictive value; ^1^: cross-validated, corresponding to the Youden index.

## Supplementary Materials

The following are available online at https://www.mdpi.com/2072-6694/11/10/1408/s1. Table S1: Clinical characteristics of patients; Table S2: Sensitivity and specificity of the genes analyzed based on the Youden index; Table S3: Prediction of lymph node malignancy in individual patients based on Model 3; Table S4: Primers and probes sequences.
